# *EagleImp*: fast and accurate genome-wide phasing and imputation in a single tool

**DOI:** 10.1093/bioinformatics/btac637

**Published:** 2022-09-20

**Authors:** Lars Wienbrandt, David Ellinghaus

**Affiliations:** Institute of Clinical Molecular Biology, Kiel University, 24105 Kiel, Germany; Institute of Clinical Molecular Biology, Kiel University, 24105 Kiel, Germany; Novo Nordisk Foundation Center for Protein Research, Disease Systems Biology, Faculty of Health and Medical Sciences, University of Copenhagen, 2200 Copenhagen, Denmark

## Abstract

**Motivation:**

Reference-based phasing and genotype imputation algorithms have been developed with sublinear theoretical runtime behaviour, but runtimes are still high in practice when large genome-wide reference datasets are used.

**Results:**

We developed *EagleImp*, a software based on the methods used in the existing tools *Eagle2* and *PBWT*, which allows accurate and accelerated phasing and imputation in a single tool by algorithmic and technical improvements and new features. We compared accuracy and runtime of *EagleImp* with *Eagle2*, *PBWT* and prominent imputation servers using whole-genome sequencing data from the 1000 Genomes Project, the Haplotype Reference Consortium and simulated data with 1 million reference genomes. *EagleImp* was 2–30 times faster (depending on the single or multiprocessor configuration selected and the size of the reference panel) than *Eagle2* combined with *PBWT*, with the same or better phasing and imputation quality in all tested scenarios. For common variants investigated in typical genome-wide association studies, *EagleImp* provided same or higher imputation accuracy than the *Sanger Imputation Service*, *Michigan Imputation Server* and the newly developed *TOPMed Imputation Server*, despite larger (not publicly available) reference panels. Additional features include automated chromosome splitting and memory management at runtime to avoid job aborts, fast reading and writing of large files and various user-configurable algorithm and output options. Due to the technical optimizations, *EagleImp* can perform fast and accurate reference-based phasing and imputation and is ready for future large reference panels in the order of 1 million genomes.

**Availability and implementation:**

*EagleImp* is implemented in C++ and freely available for download at https://github.com/ikmb/eagleimp.

**Supplementary information:**

Supplementary data are available at *Bioinformatics* online.

## 1 Introduction

Genotype phasing and imputation have become standard procedures to improve statistical power in *genome-wide association studies (GWAS)* by increasing genome-wide coverage of variants and enabling meta-analysis of individual GWAS studies. In general, the accuracy of phasing and imputation increases with the number of haplotypes from a reference panel of sequenced genomes (McCarthy *et al.*, 2016). Due to the algorithmic complexity of the imputation process a larger reference panel implies an increase in runtime and memory demand, depending on the number of unique haplotypes in each genomic segment of the target samples (e.g. in the GWAS input dataset) and the total number of these segments in the reference panel. State-of-the-art reference-based phasing and imputation algorithms such as *Eagle2* (Loh *et al.*, 2016), *PBWT* (Durbin, 2014) and *minimac4* (Das *et al.*, 2016; Fuchsberger *et al.*, 2014) have been efficiently developed with runtimes better than linear scaling over the size of the reference panel. For example, Das *et al.* (2016) showed that increasing the reference panel size from 1092 (1000 Genomes Project Phase 1, 27 million variants) to 33 000 individuals [*Haplotype Reference Consortium (HRC)*, 40 million variants] (>40-fold increase in the number of reference genotypes) only increases the phasing and imputation runtime by a factor of 10 (5.3 h versus 51.3 h) to impute 100 GWAS samples in one single-threaded process. However, for a new reference panel with more than 1 million reference samples, this would still result in a runtime of weeks, combined with an extremely increasing system memory demand. The *UK Biobank (UKB)* has recently made *whole-genome sequencing (WGS)* data from 200 000 individuals available (Bycroft *et al.*, 2018), and the newly established *European ‘1+ Million Genomes’ Initiative (1+MG)* project is underway, generating WGS data of over 1 million genomes (https://digital-strategy.ec.europa.eu/en/policies/1-million-genomes). The 1+MG project will lead to a further increase in the number of reference genotypes by more than >30-fold compared to the HRC panel benchmarked by Das *et al.* (2016). Currently, the best solution to perform phasing and imputation for large datasets is to perform parallel processing on large multi-core systems or *high-performance computing (HPC)* clusters with hundreds of CPU-cores, to distribute the computational load as much as possible. Therefore, algorithmic and technical improvements together with existing implementations are needed to ensure that phasing and imputation remain feasible for reference panels with more than 1 million samples.

## 2 Approach

Since early studies have shown that the accuracy of imputation increases significantly when the genotype data contains information on the haplotype phase of heterozygous variants (Browning, 2008), it is common practice to apply a haplotype phasing algorithm to a target input dataset prior to genotype imputation. Among others, the best known phasing tools are *SHAPEIT2* (Delaneau *et al.*, 2013), *SHAPEIT4* (Delaneau *et al.*, 2019) and *Eagle2* (Loh *et al.*, 2016), whereby the latter is currently used on all prominent imputation servers, such as the *Sanger Imputation Service (SIS)* (McCarthy *et al.*, 2016) (https://imputation.sanger.ac.uk), *Michigan Imputation Server (MIS)* (Das *et al.*, 2016) (https://imputationserver.sph.umich.edu) and the newly developed *TOPMed Imputation Server (TOPMed)* (Taliun *et al.*, 2021) (https://imputation.biodatacatalyst.nhlbi.nih.gov). Prominent imputation tools are *IMPUTE v2* (Howie *et al.*, 2009), *minimac4* (Das *et al.*, 2016; Fuchsberger *et al.*, 2014), *Beagle 5.0* (Browning *et al.*, 2018) and *PBWT* (Durbin, 2014), whereby the latter is used within the *SIS*.

To allow phasing and imputation for very large reference panels, while ensuring at least the same phasing and imputation accuracy, we developed *EagleImp*, a software tool for accelerated phasing and imputation. *EagleImp* introduces algorithmic, parameter and implementation improvements to the core methods of the established tools *Eagle2* (Loh *et al.*, 2016) (phasing) and *PBWT* (Durbin, 2014) (imputation) and merges them in a new single convenient application. With *EagleImp*, we were able to speed up the classical 2-step imputation process with *Eagle2* and subsequent *PBWT* by more than a factor of ten for single chromosome analysis and (depending on the parallelization strategy) at least more than a factor of two for the entire human genome while maintaining or even improving phasing and imputation quality. For a simulated reference panel with 1 million samples, we achieved speedups of at least 6.82 and 30.6, respectively, with phasing and imputation of a GWAS dataset using *EagleImp* compared to the phasing-only process in *Eagle2* (subsequent imputation of phasing results from *Eagle2* using *PBWT* was not possible due *PBWT*’s high memory demand, which exceeded 256 GB of system memory).

*EagleImp* also provides new convenient features via simple command line parameters, such as a continuous progress report to a file, user pre-selection of per genotype imputation information (genotypes, allele dosage, genotype dosage and/or probabilities), phasing confidences and usage information of input variants in a separate file, variant IDs from the reference in imputation output, automated chromosome chunking (if main memory requirements are too high), lock file support (to enable two or more processes to share CPU resources), detection and handling of ref/alt swaps and/or strand flips, the ability to skip certain parts of the algorithm (e.g. pre-phasing, reverse phasing or entire phasing or imputation) and more. In addition, *EagleImp* supports imputation of chromosome X and Y with automatic partitioning by pseudo-autosomal regions (*PAR*).

## 3 Materials and methods

In developing *EagleImp*, we focused on combining and improving the core methods from *Eagle2* and *PBWT*, since both tools are used by the established *SIS* web service and both use the same-named *Position-based Burrows-Wheeler Transform (PBWT)* data structure introduced by Durbin (2014), which we could target as a basis for acceleration. Its main advantages are the compact representation of binary data and the ability to quickly look up any binary sequence at any position in the data. The runtime complexity is linear to the length of the query sequence, independent of the size of the database. To create a PBWT, the algorithm determines permutations of the input sequences for each genomic site such that the subsequences ending at that site are sorted when read backwards. In our work, we propose further algorithmic and implementation improvements that allow a more efficient use of the PBWT data structure and thus increase the speed of phasing and imputation, while maintaining at least the same accuracy of phasing and imputation.

### 3.1 Improvements in *EagleImp*

For algorithmic and computational details of the original phasing in *Eagle2* and imputation in *PBWT*, we refer to our Supplementary Material S1 and the original publications by Loh *et al.* (2016) and Durbin (2014). Full details of *EagleImp* improvements summarized below can be found in the Supplementary Material S2.

**Fig. 1.**
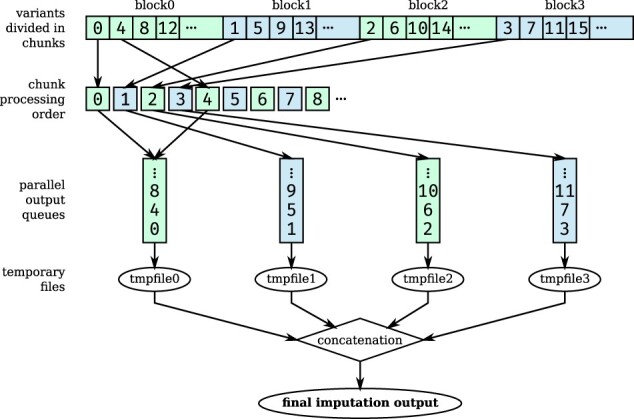
Newly implemented multi-processing scheme for imputation with *EagleImp*. After calculating set-maximal matches by intuitively distributing all target samples over multiple threads, variants to be imputed are evenly distributed over blocks with a separate output file assigned to each block. Each block is further divided into chunks that are processed in the indicated order by repetitively iterating over the blocks. The output files are written concurrently with one thread assigned to each output file. The processing of each chunk is multi-threaded over all samples using the remaining available system threads. Output files are concatenated at the end. The example shows a distribution over four blocks. The numbers in the chunks indicate the order in which the chunks are processed. See Supplementary Material S2.3.5 for details

First, the following points summarize the *EagleImp* improvements to the data structure and further technical improvements: (i) we have developed a new .qref format for reference data, which significantly improves the reading time of the reference data (Supplementary Material S2.1). (ii) The PBWT data structure of the *condensed reference* (Supplementary Material S1.1) required for each target sample is now stored in a compressed format (Supplementary Material Fig. S1 in Supplementary Material S2.2), i.e. a binary format (in permuted form) corresponding to the calculated permutation arrays with an index similar to the *FM-index* used for a *Burrows-Wheeler transformation (BWT)* (Ferragina and Manzini, 2000) (Supplementary Material Listing S1), to ensure fast generation, compact storage and fast access to the reference data. (iii) Haplotype probabilities are no longer stored in a log-based format and a non-normalized scaling factor is used for the haplotype path probabilities (Supplementary Material S2.2), which only needs to be updated in case of a predictable loss of precision after several path extension operations. In this way, probability calculations remain precise, especially for heavily needed summations of floating point numbers without an otherwise required approximation (as in *Eagle2*) or back-transformation. (iv) The imputation of missing genotypes during phasing is obsolete since the subsequent imputation step imputes missing genotypes for shared variants (between target and reference) in the same way as variants that only occur in the reference. To implement this, we used a tree structure to calculate set-maximal matches (Supplementary Material S2.3). (v) Unlike the original *PBWT* tool, *EagleImp* uses multiple threads for genotype imputation, including the use of multiple temporary output files to reduce the input/output file bottleneck (Fig. 1 and Supplementary Material S2.3). (vi) We introduced a conversion of genotypes and haplotypes into a compact representation with integer registers and made extensive use of Boolean and bit masking operations as well as processor directives for bit operations (such as *popcount* for counting the set bits in a register) throughout the application which accelerated the computing time significantly (Supplementary Material Fig. S1 and Supplementary Material Listing S2).

**Fig. 2.**
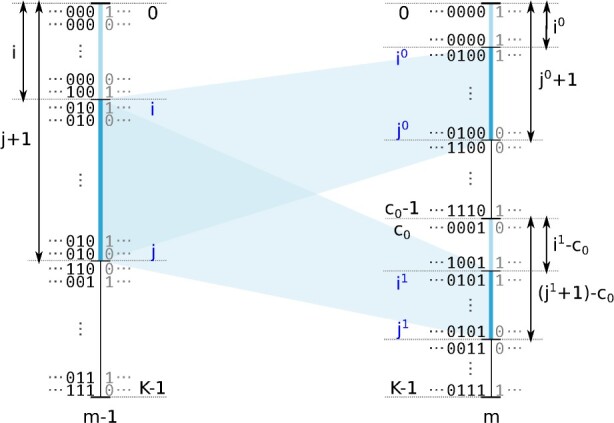
Improved path extension step in *EagleImp*. Illustration of mapping a PBWT interval ${\left[i,j\right]}_{m-1}$ from position *m −* 1 to *m*, resulting in two intervals ${\left[i^{0},j^{0}\right]}_{m}$ and ${\left[i^{1},j^{1}\right]}_{m}$. The interval ${\left[i,j\right]}_{m-1}$ exemplary represents the sequences in the PBWT that end with 010 at position *m −* 1. By stepping forward to position *m* these sequences are extended either with 0 or 1 and thus are located in one of the mapped intervals ${\left[i^{0},j^{0}\right]}_{m}$ or ${\left[i^{1},j^{1}\right]}_{m}$, respectively. It is easy to see, that $i=i_{0}+(i_{1}-c_{0})$ and $j+1=(j_{0}+1)+((j_{1}+1)-c_{0})$ (as *j* is inclusive in the interval) which directly leads to the mapping Equations (6) and (7) in Supplementary Material S2.1

Second, the following points summarize further algorithmic improvements in *EagleImp*: (i) due to the properties of the PBWT data structure, sequences that end with equal subsequences at a certain position are located next to each other in the PBWT and can thus be addressed as an *interval*. For the path extension step now only the interval boundaries have to be mapped to the next position to get the corresponding intervals for both possible extensions of the sequence (Fig. 2 and Supplementary Material Listing S2). Since the frequency of a subsequence is equal to the (normalized) size of the corresponding interval, the frequency calculation could thus be accelerated. (ii) We increased the default beam width parameter (number of possible paths) from 50 (fixed value in *Eagle2*) to 128 and the Δ parameter from 20 (fixed value in *Eagle2*) to 24 in favor of further increasing the phasing quality with minimal loss of computing time (Supplementary Material S1.1 and S2.2). (iii) We omitted the *identical-by-descent (IBD)* check performed by *Eagle2* before the phasing process, since we found no loss in phasing quality without the IBD-check implemented. (iv) Pre-phasing is disabled by default as we encountered no improvement, but can explicitly be enabled with a user option in *EagleImp*. Likewise, reverse phasing can optionally be disabled.

Third, we introduced new features and improvements in usability, among other things: (i) *EagleImp* allows imputation of haploid samples on X and Y chromosomes. Especially for chromosome X imputation, the problem is that diploid data in the PAR and haploid data in the non-PAR regions occur in the same chromosome X input file. Existing tools and imputation servers often crash with an error message if diploid and haploid data is mixed in a single input file. We provide a script together with the *EagleImp* source code that takes care of these regions by automatically splitting the input data with respect to PAR regions on chromosome X before imputation and then merging the imputation results back into one file afterwards. (ii) *EagleImp* allows reference and alternative alleles in the target to be swapped compared to the reference (with the option –allowRefAltSwap), e.g. an A/C variant is considered as C/A, and it allows strands to be flipped, e.g. an A/C variant is considered a T/G variant at the same chromosomal position (with the option –allowStrandFlip). (iii) *EagleImp* computes the imputation accuracy *r*^2^ [as described in Das *et al.* (2018) and used in *minimac4*] and provides the value together with the allele frequency, the minor allele frequency (MAF), the allele count and number as well as the reference panel allele frequency (if available) in the imputation output. An optional *r*^2^ filter can be applied to filter out variants with low imputation quality. (iv) Phasing confidences and information about how the target variants are used for imputation are provided in separate output files. (v) To save disc space for the output files, the user can decide which information is provided along with the imputed (hard called) genotypes, i.e. any combination of allele dosages (*ADS* tag), genotype dosages (*DS* tag), genotype probabilities (*GP* tag) or no information. Variant IDs in imputation output are provided exactly as they appear in the reference. (vi) *EagleImp* automatically activates chromosome chunking if the memory requirement is higher than the available main memory (provided as the runtime parameter –maxChunkMem) to eliminate the tedious process of trying out chunk sizes on different input and reference datasets for the user. (vii) For better workload distribution on multi-core computers, we provide a locking mechanism (via a lock file) such that low CPU-load tasks (e.g. reading input files) can run multiple processes at once, while high CPU-load tasks (e.g. the phasing and imputation processes) require multiple-exclusion of CPU resources. We provide an optional launch script that uses this feature for simultaneous processing of multiple input files. (viii) A progress indicator shows the progress in percentage (giving the user a hint how long the analysis will take). Optionally, constantly updated status and info files display summarized information about the running process.

### 3.2 Benchmark settings

To quantify phasing and imputation quality and runtime improvements of *EagleImp* compared to the original tools *Eagle2* and *PBWT*, first, we conducted quality benchmarks with different parameters on three different sized reference panels (Table 1) and 12 target datasets (Table 2) including two real-world target GWAS datasets [from Ellinghaus *et al.* (2020)] to further compare imputation accuracy of *EagleImp* with the accuracy of current imputation servers (*SIS*, *MIS* and *TOPMed*). Then, we ran all runtime benchmarks using the *HRC.EUR* target dataset (Table 2) and the parameters used for the quality benchmarks. Full descriptions about reference and target datasets from Tables 1 and 2 and details about the setup for quality and runtime comparisons (in particular, the preparation of various multi-processor configurations) can be found in Supplementary Material S3.

**Table 1.** Three different sized reference panels (A–C) were used for phasing and imputation benchmarks

	Reference	#Variants	#Samples	Ancestry
(A)	*HRC1.1*	40.4 million	27 165	Mixed^a^
(B)	*1000G Phase 3*	84.8 million	2504	Mixed^b^
(C)	*simulated*	40.4 million	1 000 000	not applicable

a Predominantly of European descent.

b Five superpopulations by continent: African (AFR), American (AMR), East Asian (EAS), European (EUR) and South Asian (SAS).

**Table 2.** Twelve target datasets in total were used for phasing and imputation benchmarks to evaluate the runtime and imputation accuracy of *EagleImp* compared to the original tools *Eagle2* and *PBWT*

	Target	#Variants	#Samples	Ancestry
(1)	*HRC.AFR*	619 872	661	African
(2)	*HRC.AMR*	619 872	347	American
(3)	*HRC.EAS*	619 872	504	East Asian
(4)	*HRC.EUR*	619 872	494	European
(5)	*HRC.SAS*	619 872	489	South Asian
(6)	*1kG.AFR*	647 963	50	African
(7)	*1kG.AMR*	647 963	50	American
(8)	*1kG.EAS*	647 963	50	East Asian
(9)	*1kG.EUR*	647 963	50	European
(10)	*1kG.SAS*	647 963	50	South Asian
(11)	*COVID.Italy*	559 519	2113	Italian
(12)	*COVID.Spain*	549 696	1792	Spanish

*Note*: Target datasets (1–5) were sampled from the *HRC1.1* panel (A) and target datasets (6–10) were sampled from the *1000G Phase 3* panel (B). For each of the target datasets (1–10), the selected samples were removed from the corresponding reference panels (A) and (B) in the benchmarks to avoid a biased result. The real-world datasets (11–12) [from Ellinghaus *et al.* (2020)] did not show an overlap of samples with any of the reference panels. All target datasets contain data from chromosomes 1 to X.

#### 3.2.1 Choice of phasing and imputation parameters

The exact input parameters for the program call of *EagleImp*, *Eagle2* and *PBWT* are listed in Supplementary Material S3.3. For performance comparison, we focused on testing different values of the parameter *K* (to select the *K*-best haplotypes from the reference for phasing). For example, we ran a benchmark on each HRC.* dataset (Table 2 (1–5)) with four different values of *K* for *EagleImp* as well as for *Eagle2*: 10 000 (default setting in *Eagle2*), 16 384, 32 768 and ‘*max*’ {where max means using all available haplotypes for phasing, which is 54 330 minus the number of removed haplotypes [e.g. 1322 for target (1), i.e. *HRC.AFR*, corresponding to two times the number of input samples] in the case of the reduced *HRC1.1* panel (A)}. All runs used one phasing iteration (reflecting the default setting of *Eagle2* if the number of target samples is less than half of the number of reference samples).

#### 3.2.2 Benchmark system and program versions

The computing system we used for benchmarks consists of two Intel Xeon E5-2667 v4 CPUs, each with 8 cores running at 3.2 GHz, resulting in 32 available system threads. The system was equipped with 256 GB of DDR4 RAM and used a ZFS file system that combines six HDDs, each with a capacity of 2 TB in a *raidz2* pool (leading to a total capacity of about 7 TB). The operating system was Ubuntu Linux 21.10 with kernel 5.13.0.

*EagleImp* is written in C++ and compiled with GCC v11.2.0. For *Eagle2* and *PBWT*, we used the to date most recent builds on Github: *Eagle2 v2.4.1* (https://github.com/poruloh/Eagle.git) and *PBWT 3.1-v3.1-7-gf09141f* (https://github.com/VertebrateResequencing/pbwt.git). Both tools were also compiled with GCC v11.2.0 together with the required *HTSlib v1.12* and *bcftools v1.12*. For runtime benchmarks, we measured the wall-clock runtime by marking the start and end points of the benchmark with the command date and calculated the difference in runtime.

#### 3.2.3 Metrics for phasing and imputation accuracy

We define a *phase switch* whenever the current phase at a (heterozygous) call site differs from the phase at the previous call site, and we counted a *switch error* whenever a phase switch occurs at a call site in the phased target but not in the same original target sample in the reference (with the correct phase known), or vice versa, when comparing phased haplotype pairs from the target to the original haplotype pairs in the reference. The *switch error rate* per sample is then computed by dividing the switch errors by the number of target variants. Please note that switch errors in the reference cannot be completely ruled out, since the publicly available reference data was phased by algorithms themselves.

*Genotype errors* are determined in a similar way by comparing the genotypes of an imputed haplotype pair with the corresponding (correctly known) genotypes in the reference panel. However, because hard called genotypes (usually determined by a fixed allele dosage threshold of 0.5) do not properly reflect uncertainty in genotype estimations and downstream analyses often require the allele dosages instead of hard called genotypes, we calculated genotype error rates using ‘soft called’ genotypes. Therefore, the number of genotype errors for a sample is determined by $\sum_{x}\left(1.0-gp_{x}\right)$ where *x* is the correct genotype from the reference and *gp_x_* the corresponding genotype probability calculated from the imputed allele dosages. Finally, dividing by the total number of variants gives the *genotype error rate*.

Another way to determine the imputation quality is the imputation accuracy *r*^2^, which is a valuable means of interpretation with regard to sample size and statistical power in a GWAS study and which can basically be considered independent of MAF (although the precision of the *r*^2^ estimate decreases with low MAF) (Das *et al.*, 2018). *r*^2^ can be estimated for each imputed variant from posterior allele probabilities without knowing the true allele on each chromosome.

## 4 Results and discussion

### 4.1 Phasing and imputation quality

#### 4.1.1 Switch and genotype error rates

**Fig. 3.**
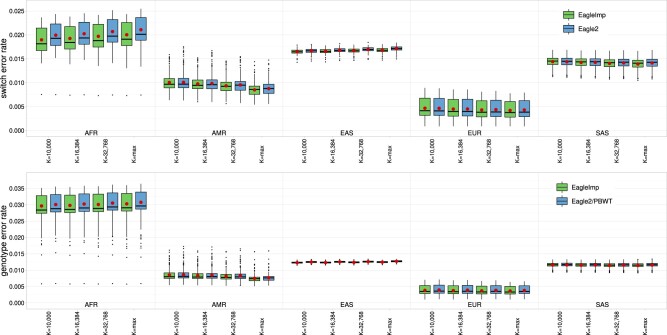
Phasing and imputation based on the *HRC1.1* reference panel [mostly European ancestry; Table 1 (A)] performed better for *EagleImp* (green) compared to the original *Eagle2/PBWT* (blue) in all five test datasets *HRC.AFR*, *HRC.AMR*, *HRC.EAS*, *HRC.EUR* and *HRC.SAS* [Table 2 (1–5**)**]. The figure shows boxplots (hinges at the first and third quartiles, whiskers extending up to 1.5× interquartile range, outliers plotted separately) for sample-wise switch error rates after phasing (top) and genotype error rates after imputation (bottom), shown for four different values of *K*: 10 000 (default setting in *Eagle2*), 16 384, 32 768 and ‘max’ (corresponding to maximum available haplotypes in reference panel). The mean values are shown as red dots. The parameter *K* selects the K-best haplotypes from the reference for phasing. As expected, phasing and imputation of input data of European ancestry using the *HRC1.1* reference panel, which consist mostly of European samples, results in the best accuracy, which increases with an increasing *K* parameter. For populations that do not match the predominant population of the reference panel, an increasing *K* parameter may lead to less accurate results. A scaled version of this figure with a stretched Y axis is provided in Supplementary Material Figure S2 to better illustrate the difference between *EagleImp* and *Eagle2/PBWT* (A color version of this figure appears in the online version of this article.)

**Fig. 4.**
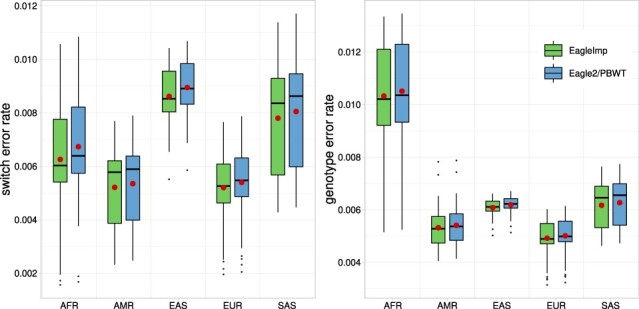
For the *1000G Phase 3* reference panel [five superpopulations; Table 1 (B)], phasing and imputation with *EagleImp* (green) outperformed the original *Eagle2/PBWT* (blue) in all five test datasets *1kG.AFR*, *1kG.AMR*, *1kG.EAS*, *1kG.EUR* and *1kG.SAS* [Table 2 (6–10)]. The figure shows boxplots (hinges at the first and third quartiles, whiskers extending up to 1.5× interquartile range, outliers plotted separately) for sample-wise switch error rates after phasing (left) and genotype error rates after imputation (right). The mean values are indicated as red dots. Parameter *K* was chosen to be maximum in all test runs (here, *K *=* *5008), thus including the entire *1000G Phase 3* reference panel (A color version of this figure appears in the online version of this article.)

For both *EagleImp* and *Eagle2/PBWT*, average (genome-wide) switch error rates and genotype error rates decreased (as expected) with higher values of *K* using the *HRC1.1* reference panel for American (AMR) and European (EUR) superpopulation datasets (Fig. 3; Supplementary Material Tables S1 and S2; Supplementary Material Fig. S2). In contrast, the African (AFR) and East Asian (EAS) populations showed an increase of the error rates with a higher *K* value which we attribute to the predominantly European ancestry of the samples in the *HRC1.1* panel (and thus the inclusion of more samples into the phasing set that do not match the target population). For the South Asian (SAS) dataset, the switch error rate decreased with a higher *K* but the genotype error rate stagnated. Given the predominantly European ancestry of the samples in the panel, our expectation that the European (EUR) dataset would perform best was also confirmed, followed by the American (AMR) dataset. Genotype error rates were more than eight times higher for the African (AFR) dataset than for to the European (EUR) dataset, while the switch error rates where about 4 times higher. In a direct comparison of *EagleImp* to *Eagle2* combined with *PBWT*, we observed that *EagleImp* performed comparably or slightly better at *K *=* *10 000 (depending on the superpopulation), with average change in switch error rates ranging from a minimal increase of 0.4% (EUR and SAS datasets) to a reduction of 4.9% (AFR dataset), and a reduction in the average genotype error rate from 0.3% (SAS dataset) to 2.6% (EUR dataset). At higher values of *K*, *EagleImp* differed more from *Eagle2/PBWT* as *EagleImp* runs performed better compared to the corresponding *Eagle2/PBWT* runs with the same value of *K*. The reduction in average switch error rates ranged from 0.1% (SAS dataset, *K *=* *16 384) to 5.0% (AFR dataset, K=max) and the reduction in genotype error rates ranged from 0.5% (SAS dataset, *K *=* *16 384) to 4.2% (EUR dataset, K=max). In our *1000G Phase 3* reference benchmark analysis (Fig. 4, Supplementary Material Tables S3 and S4), *EagleImp* performed better than *Eagle2/PBWT* for all five target datasets from the *1000G Phase 3* panel. Phase switch errors rates improved from 2.6% (AMR dataset) up to 7.1% (AFR dataset), and genotype error rates improved from 1.6% (SAS dataset) up to 1.8% (AFR, EAS and EUR datasets).

#### 4.1.2 Imputation accuracy *r*^2^

For the real-world GWAS datasets *COVID.Italy* and *COVID.Spain*, we determined *r*^2^ values stratified by MAF (including all variants above this threshold) using the four different *K* parameters from above (Supplementary Material Figs S3 and S4). The *COVID.Spain* dataset showed a generally slightly better imputation performance than the *COVID.Italy* dataset in all runs, possibly due to a more similar genetic background compared to the *HRC1.1* reference panel. Already for *K *=* *10 000 (Supplementary Material Figs S3a and S4a), phasing and imputation with *EagleImp* consistently produced higher *r*^2^ values than the *Eagle2/PBWT* combination across the entire MAF spectrum, and also higher *r*^2^ values than the *SIS*, despite its larger (not freely available) *HRC1.1* reference panel as compared to our *HRC1.1* benchmark panel. With *K *=* *10 000 and a MAF >0.002 (and especially at higher MAF), *EagleImp* also showed a quality advantage in contrast to the *MIS*, which we attribute to our algorithmic changes. Below a MAF of 0.002, the MIS still seems to show its higher imputation quality due to the larger *HRC1.1* reference panel. Especially, in this low frequency range, *TOPMed* imputation (as a reference model) shows that a much larger reference panel plays a another key role in increasing imputation accuracy for rare variants, in addition to algorithmic improvements. Interestingly, for *K *=* *10 000 and common variants such as in GWAS studies, *EagleImp* was shown to achieve even higher quality than *TOPMed* (here for MAF >0.03 for *COVID.Spain* and >0.006 for *COVID.Italy*), which is probably due to the algorithmic improvements of *EagleImp*, although *EagleImp’*s reference panel is more than three times smaller than that of *TOPMed*.

**Fig. 5.**
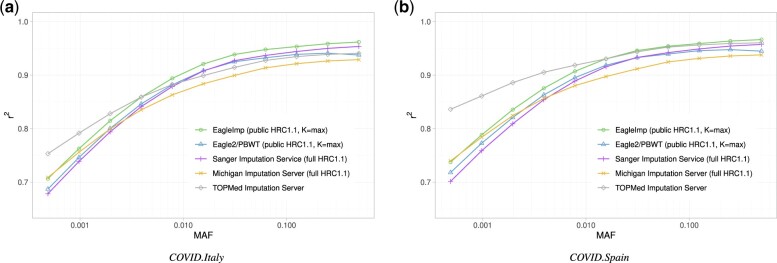
Imputation accuracy *r^2^* was higher for *EagleImp* [green; *K *=* *54 330 (max)] compared to *Eagle2/PBWT* [blue; *K *=* *54 330 (max)] for two real-world GWAS datasets (a) *COVID.Italy* and (b) *COVID.Spain* [Table 2 (11–12)], with further comparison to imputation *r*^2^ from imputation servers *SIS* (purple), *MIS* (orange) and *TOPMed* (grey). *EagleImp* showed a quality gain compared to *TOPMed* for minor allele frequency (MAF) >0.004 (*COVID. Italy*) and 0.01 (*COVID.Spain*) and achieved at least equivalent results compared to *MIS* across the entire MAF spectrum, although *MIS*/*SIS* and *TOPMed* use their own reference panels, which are ∼1.2 times (32 470 samples) and 3.5 times (97 256 samples) larger than the publicly available *HRC1.1* panel [27 165 samples; Table 1 (A)] used for *EagleImp* and *Eagle2/PBWT*. Note that the *TOPMed* reference panel contains 186 million variants in contrast to the 40 million variants of the *HRC1.1* panel, and we did not apply an *r*^2^-filter (A color version of this figure appears in the online version of this article.)

For higher *K* parameters, the graphs for *EagleImp* and *Eagle2/PBWT* showed (as expected) better *r*^2^ values but the distance between both tools remained the same (Supplementary Material Figs S3b–d and S4b–d). Figure 5 depicts the average genome-wide *r*^2^ values for the *COVID.Italy* and *COVID.Spain* datasets as a function of different MAF thresholds (on logarithmic scale), for *Eagle2* and *EagleImp* with K=max (which is effectively *K *=* *54 330 as we used the public *HRC1.1* panel). Here, *EagleImp* achieved at least equivalent or better results compared to the *MIS* across the entire MAF spectrum and showed same quality or a quality gain compared to *TOPMed* for MAF >0.01 (*COVID.Spain*) and 0.004 (*COVID.Italy*). Please note that we did not apply any *r*^2^-filter and that the *TOPMed* results contain more than 186 million variants (in comparison to 40 million from the *HRC1.1* panel). Also note, that because MIS and TOPMed do not provide reference panel MAFs, the study data set MAFs are presented here with a MAF cut-off of 0.0005 to ensure a sufficiently high estimate of the MAF of the imputed variants for the input study datasets. However, the same behaviour is visible, although not as clearly, in the visualizations using the reference panel MAF (RefPanelMAF) (Supplementary Material Figs S5 and S6—reference panelMAFs for the MIS were taken from the SIS (due to unavailability from MIS), and the *TOPMed* results could not be shown due to unavailability of reference panel MAFs from *TOPMed*).

### 4.2 Phasing and imputation runtime

For the sake of simplicity, we ran all runtime benchmarks using the *HRC.EUR* target dataset and the parameters used for the *HRC1.1* reference benchmarks above, but with different multi-processing configurations with up to 32 concurrent system threads for *EagleImp* and *Eagle2/PBWT*. Details of the various multiprocessor configurations tested can be found in Supplementary Material S3.6. In addition, we examined runtimes of individuals chromosomes processed with all 32 system threads (referred to as *1x32* runs) (Supplementary Material S3.7) and single-thread runtimes with multi-processing disabled (Supplementary Material S3.8), since multi-processing strategies can behave differently on different computing systems. We also measured the runtimes of our real-world GWAS datasets *COVID.Italy* and *COVID.Spain* which can be found in Supplementary Material S3.9. Note, that the runtime benchmarks do not include the preparation of the reference files, which is required for *PBWT* and is optional for *EagleImp*. (*PBWT* requires a .pbwt file for each reference file; for *EagleImp* we used our newly developed .qref format instead of .vcf.gz or .bcf.) We present the runtimes for converting the reference panel separately in Supplementary Material S3.10.

#### 4.2.1 Multi-processing runtimes

We observed that for any value of *K*, the *Eagle2.8x4* configuration performed best for the *Eagle2/PBWT* runs and the *EagleImp.2x4x8* configuration performed best for *EagleImp* (Supplementary Material Table S5), which is why we only compared these two configurations in the following.

**Fig. 6.**
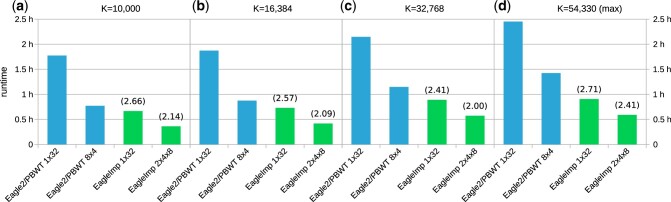
Faster runtimes of *EagleImp* compared to *Eagle2/PBWT* (with at least the same accuracy, Fig. 3), demonstrated for the processing of 494 European target samples using the *HRC1.1* reference panel [Table 1(A)] and different *K* parameters (*K *=* *54 330 denotes the maximum value including all haplotypes from the HRC1.1 panel). The naive multi-processor configuration (*1x32* with 32 phasing threads and each chromosome processed sequentially) and the best individual multi-processor configuration (i.e. fastest individual configuration determined from six different runs of *Eagle2/PBWT* and eight different runs of *EagleImp*, see Supplementary Material Table S5) were compared. The numbers in brackets give the acceleration factor of *EagleImp* compared to the corresponding *Eagle2/PBWT* run

For *K *=* *10 000 (Fig. 6a), *Eagle2.1x32* took 1 h and 46 min: The *Eagle2.8x4* configuration accelerated this by a factor of 2.30 to 46 min. In contrast, the *EagleImp.1x32* configuration required 40 min, which we could speed up by a factor of 1.85–22 min in the *EagleImp.2x4x8* configuration. This corresponds to a speedup factor of 2.66 when comparing the *1x32* configurations of *Eagle2/PBWT* and *EagleImp* or a factor of 2.14 when comparing the fastest multi-processing configurations of the two tools. The total runtime increased with higher values of *K* (Fig. 6b–d), but the speedup factors between both tools only varied slightly. For K=max, the *Eagle2/PBWT* runtime was 2 h and 27 min for the *1x32* configuration and 1 h and 25 min for *8x4*. *EagleImp* analysed the same data in 54 min (*1x32*) and 35 min (*2x4x8*), resulting in speedup factors of 2.71 and 2.41, respectively.

When comparing runtimes for individual chromosomes, we additionally measured runtimes for phasing-only, imputation-only and combined runs. For the phasing-only runs, we observed that *EagleImp* is between 2.93 and 4.81 times faster than *Eagle2* for all chromosomes and different values of *K* (Supplementary Material Table S6). The imputation-only runs of *EagleImp* with 32 threads when compared to *PBWT* showed the advantages of the multi-threading capability of *EagleImp* (which *PBWT* does not offer), with a speedup between 7.77 and 12.94 (Supplementary Material Table S7). The combination of phasing and imputation offers the advantage for *EagleImp* that the reference panel does not have to be read twice (as is the case with *Eagle2* and *PBWT*), resulting in a combined speedup between 5.82 and 10.81 for single chromosomes (Supplementary Material Table S8).

#### 4.2.2 Single-thread runtimes

We measured the runtimes of the *HRC.EUR* dataset from above with the four values of *K* exemplary for chromosome 2 and chromosome 21 (largest and smallest number of input variants) with only one thread for *Eagle2* and *EagleImp* (by using the corresponding –numThreads parameters for both tools). We found that *EagleImp* is faster than *Eagle2/PBWT* with measured speedups between 1.59 and 2.48 for phasing only and between 1.40 and 1.51 for imputation only, and a combined speedup between 1.58 and 2.25 (Supplementary Material Tables S9 and S10).

#### 4.2.3 Phasing and imputation using 1 million reference genomes

To our knowledge, the *TOPMed* imputation server uses the largest reference panel currently available for phasing and imputation, with 97 256 reference samples (*TOPMed r2*) (Taliun *et al.*, 2021). To explore *EagleImp’*s capabilities in handling a future reference panel with way more samples, we created a simulated panel [Table 1 (C); Supplementary Material S3.1] containing 1 million samples and 40.4 million variants (taken from the *HRC1.1* panel) by generating random haplotypes for each variant while maintaining the reference panel allele frequency of the original *HRC1.1* panel. We measured *EagleImp’*s and *Eagle2*’s runtimes for the real-world GWAS dataset *COVID.Italy* (2113 samples) using *K *=* *10 000 and *K *=* *2 000 000 (maximum *K* for the simulated panel) and the naive (*Eagle2.1x32*, *EagleImp.1x32*) and respectively fastest multi-processor configurations (*Eagle2.8x4*, *EagleImp.2x4x8*) from the previous runtime benchmark (Supplementary Material S3.11).

**Fig. 7.**
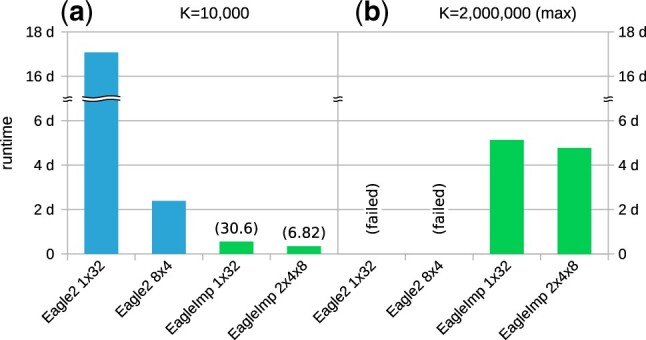
Phasing and imputation runtimes for the simulated reference panel [Table 1 (C)] containing 1 million samples (2 000 000 haplotypes) and the real-world *COVID.Italy* dataset [2113 samples; Table 2 (11)]. The numbers in brackets give the acceleration factor of *EagleImp* (phasing + imputation) compared to the corresponding *Eagle2* (phasing only) run. Note that we could not perform imputation with *PBWT* using this panel due to insufficient main memory to generate the required .pbwt files, so only *Eagle2* phasing times are shown. For K=max*Eagle2* could not complete the analysis in any configuration due to insufficient main memory again (marked with ‘failed’), hence, no runtime or speedup could be measured in this configuration

*EagleImp* took about 8 h and 25 min for *K *=* *10 000 in *2x4x8* configuration for phasing and imputation, while, in contrast, the original *Eagle2* took more than 2 days and 9 h in *8x4* configuration respectively for phasing only. Thus, *EagleImp* outperformed *Eagle2* with a speedup factor of at least 6.82 (*K *=* *10 000, Fig. 7). For the naive configuration (*1x32*) *Eagle2* took even more than 17 days for the analysis, which is a speedup of 30.6 for *EagleImp* that took only less than 14 h. Note that when using the smaller *HRC1.1* reference panel for the same benchmark *Eagle2* took 1 h and 26 min for phasing alone while *EagleImp* took only 40 min for phasing and imputation together, which is a speedup of 2.16 already (see Supplementary Material Tables S5 and S6). This highlights that runtime benefits of *EagleImp* grow with an increasing size of the reference panel. The runs with K=max, i.e. *K *=* *2 000 000 in this case, resulted in a runtime of less than 4 days and 19 h for *EagleImp* in *2x4x8* configuration, while *Eagle2* could not complete the runs due to insufficient memory and missing automatic chunking ability (requirement of more than 256 GB of RAM) on our benchmark system in both *8x4* and *1x32* configurations. Note that we needed to compare *EagleImp* runs including phasing and imputation to *Eagle2* runs with phasing only as we were not able to run a *PBWT* imputation afterwards. *PBWT* requires the reference panel converted to a .pbwt format which failed due to insufficient main memory again.

## 5 Conclusion

We have introduced *EagleImp*, a fast and accurate tool for phasing and imputation. Due to technical improvements and changes in the data structure, *EagleImp* was 2 to 30 times faster (depending on the reference panel size and multi-processor configuration) than *Eagle2/PBWT*, with same or better phasing and imputation quality in all tested scenarios. For common variants investigated in typical GWAS studies, *EagleImp* also yielded equal or higher imputation accuracy than the imputation servers *MIS*, *SIS* and *TOPMed* that use larger (not freely available) reference panels, which we attribute to our algorithmic improvements. Because of the technical optimization and the improvement of the stability of the software, *EagleImp* can perform phasing and imputation for upcoming very large reference panels with more than 1 million genomes.

For phasing, we accelerated the search for the *K* best haplotypes, the generation of the condensed reference and the entire phasing process by using an alternative haplotype encoding, Boolean comparison operations and processor directives for bit operations. We improved the accuracy of the phase probability calculation by using a scaled floating point representation instead of a logarithm-based representation, and we improved the frequency lookups in the PBWT data structure by introducing our interval mapping procedure and omitting duplicate calculations of frequencies. By adding a multi-threading scheme that includes concurrent writing of multiple temporary files during imputation, and by using a tree-structure and the same interval mapping technique as for the phasing part to search for set-maximal matches, we were able to speed up the imputation process considerably compared to *PBWT*. Furthermore, the correct treatment of missing genotypes from the input dataset became possible with the tree-structure.

An additional reduction in computing time was made possible by the introduction of the *Qref* -format (producible from standard .vcf or .bcf files) for fast reference loading, by combining phasing and imputation in a single tool, and by a helper script that introduces multi-processing with several worker processes and a balanced distribution of a complete genome-wide input dataset. We further enhanced the usability of phasing and imputation by adding several convenience features, such as chromosome X and Y handling, imputation *r*^2^ and *MAF* calculation for output files, user selection of desired imputation information (allele dosages, genotype dosages, genotype probabilities), automated optimization of chromosome partitioning (chunking) and memory management during runtime, preserving variant IDs from reference, *r*^2^ filtering and other things.

Unfortunately, we were not able to sufficiently investigate the quality of the imputation of rare variants, as we did not have the larger reference panels of the *MIS*, *SIS* and the *TOPMed* imputation servers at our disposal. We noticed that in the imputation of common variants, the *MIS* (uses *minimac4* for imputation) fell behind the *SIS* (uses *PBWT* for imputation) in terms of quality, despite the same larger HRC reference panel, but the *MIS* performed better than the *SIS* in the imputation of rare variants. We have not investigated this further. However, since new, even larger reference panels will be available soon, this difference should not matter too much. We already designed *EagleImp* to use more than 1 million reference genomes (>2 million haplotypes) for reference-based phasing and imputation. In this case, *EagleImp* will be able to show its full strength in terms of runtime and quality compared to other tools, although again an optimization of the *K* parameter (selection of the *K* best reference genomes where a higher *K* increases the runtime but produces better results) will be required. Imputation in combination with new methods such as study-specific pre-selection of reference samples using deep learning imputation reconstruction methods for reference panels (Shi *et al.*, 2021) could provide a higher accuracy for rare variants.

As an improvement, in the future, we plan to replace the slow *HTSlib* with our own library in order to speed up the writing process since this is still a bottleneck in *EagleImp*. A reduction of the runtime by using *field-programmable gate arrays (FPGAs)* is another possibility to reduce the runtime even further (Wienbrandt *et al.*, 2020).

Since the existing imputation servers do not all offer the same reference panels (*1000 Genomes*, *HRC*, *TOPMed*) for different imputation scenarios (e.g. imputation of individuals of non-European descent) and also the topic of the European Union (EU) *General Data Protection Regulation (GDPR)* is not sufficiently addressed in our view, it would also be desirable to set up mirror servers in different countries and to provide them with the same algorithms and reference panels to facilitate comparability of imputation results as well as to allow optimization of phasing and imputation for different GWAS input data sets, for example, by appropriate choices of input parameters. For example, the *K* parameter basically increases the accuracy in case of high similarity between input and reference, but may also decrease the accuracy in case of low similarity as we observed e.g. when imputing the *HRC.AFR* dataset of African origin with the *HRC1.1* reference of predominantly European origin. Thus, we plan to offer a free GDPR-compliant web service for *EagleImp* with adjustable input parameters (e.g. the *K* parameter), so that the advantages of *EagleImp* can also be used by the community even without own hardware resources.

## Availability of source code and requirements

- Project name: EagleImp
- Project home page: https://github.com/ikmb/eagleimp
- Operating system: Linux
- Programming language: C++ (bash, awk)
- Other requirements: HTSlib, Zlib, BOOST, TBB, Cmake
- License: GNU GPL v3.0

## Ethical approval

GWAS data from the COVID-19 GWAS Group: All participants provided written informed consent, and the study was approved by the ethics boards of the participating institutions in agreement with the Declaration of Helsinki principles.

## Supplementary Material

btac637_Supplementary_Data

## Data Availability

All quality and runtime measures from our benchmarks are listed in the Supplementary Material. The *1000 Genomes Phase 3* reference panel can be downloaded at ftp://ftp.1000genomes.ebi.ac.uk/vol1/ftp/release/20130502/. Data access to the EGAD00001002729 dataset for the HRC1.1 panel is restricted and was granted under request ID 11699. The benchmark datasets *HRC.EUR*, *HRC.v1–5*, *1kG.EUR.v1–5* and *1kG.v1–5* are subsets of the previously mentioned reference panels. The COVID-19 GWAS datasets *COVID.Italy* and *COVID.Spain* used in this article cannot be shared publicly due to the privacy of individuals that participated in the study. For further information on these datasets we kindly refer to Ellinghaus *et al.* (2020).
